# Variable deep learning training horizons reveal the temporal complexity of biological systems

**DOI:** 10.17912/micropub.biology.001926

**Published:** 2026-02-18

**Authors:** Po-Hao Chiu, Jacob I Evarts, Patrick Feng, Neda Bagheri

**Affiliations:** 1 Chemical Engineering, University of Washington, Seattle, Washington, United States; 2 Biology, University of Washington, Seattle, Washington, United States

## Abstract

The increasing quantity of time-series images presents new opportunities for extracting biological insights from data. Here, we introduce a deep learning framework with a variable input sequence length to predict cell and colony morphologies. We apply this framework to *in silico* and *in vitro* microscopy datasets, evaluating the impact of temporal data on performance. We find that while performance increases monotonically with increasing *in silico* training data, performance is varied in the *in vitro* case studies. The varying results reflect the intrinsic challenges stochastic, complex biological systems pose to data-driven modeling, and offer a new method through which we can identify biological transition points using temporal dynamics.

**Figure 1. FNOs trained on varying temporal input horizons exhibit different performance outcomes:**
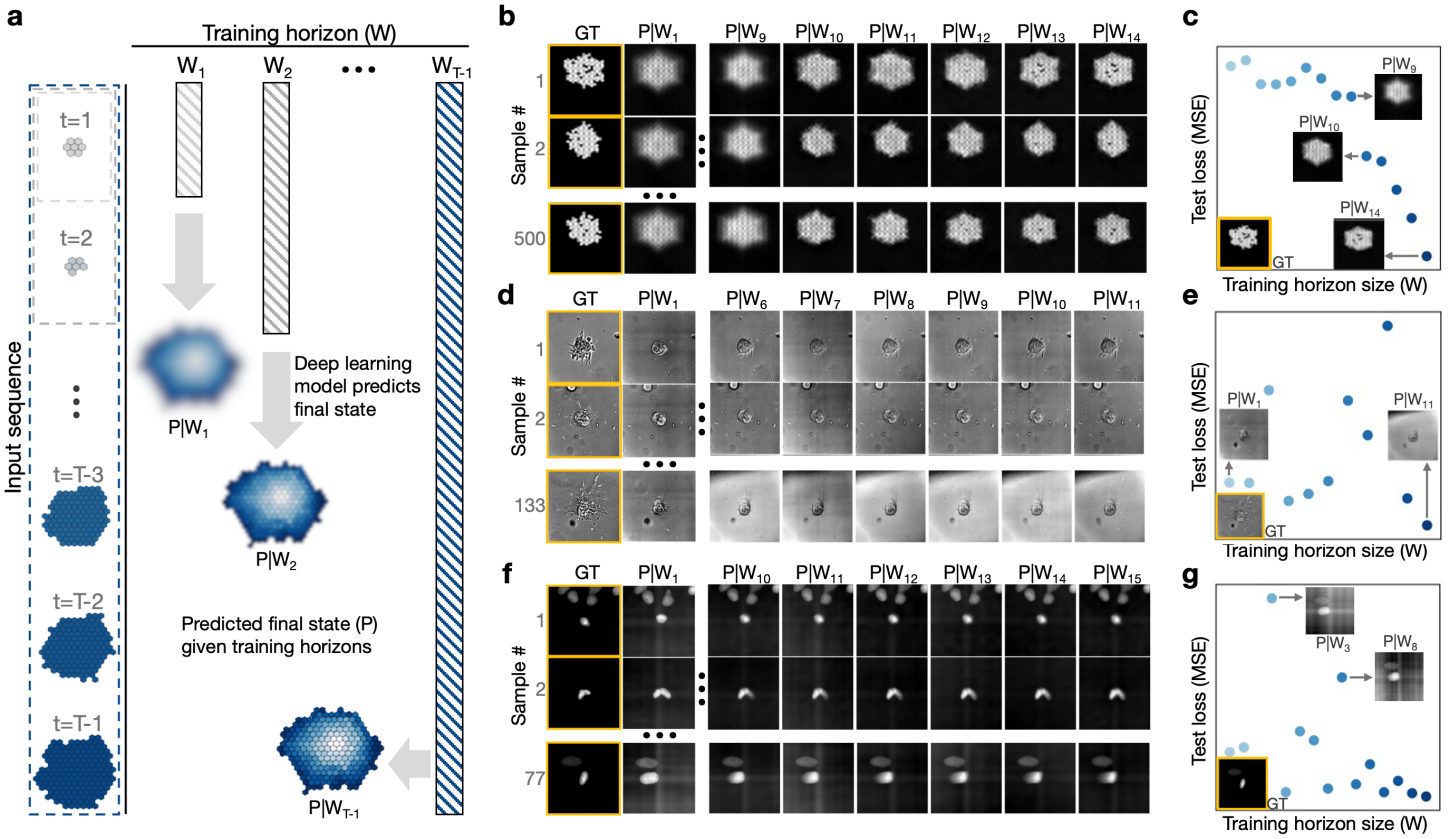
( **a** ) The deep learning pipeline enables interrogation of the impact of the training window horizon on prediction accuracy. An ARCADE simulation sequence is shown with gradient colors that correspond with tumor age. A variable input window W _i_ is passed through an FNO that is trained to predict the final timepoint of the time series, which we term the prediction horizon P. ( **b** ) FNO predictions based on increasing training horizons are shown for three of 500 samples of the in silico tumor simulation data. The FNO predicts images at the 15th time point based on an input window W _i_ where 1≤i≤14. The leftmost column shows the ground truth image highlighted in yellow, and each subsequent column shows the predicted image given a window length W _i_ . Each row corresponds to a unique sample. ( **c** ) The test loss (quantified by the mean squared error, MSE) of *in silico* simulation data is shown as a function of training window size. Results highlight nearly monotonic improvement of performance with increased training window horizon. The image in the bottom left corner is the ground truth of sample 1 in panel b (highlighted in yellow), and notable predictions are shown for select window horizons. ( **d** ) FNO predictions based on increasing training horizons are shown for three of 133 samples of the in vitro tumor spheroid data. The FNO predicts images at the 12th time point based on an input window W _i_ where 1≤i≤11. The leftmost column shows the ground truth image highlighted in yellow, and each subsequent column shows the predicted image given a window length W _i_ . Each row corresponds to a unique sample. ( **e** ) The test loss (MSE) of *in vitro* spheroid images is shown as a function of training window size. Unlike the simulated tumor case study, there are considerable fluctuations in FNO performance even with an increase in window horizon and temporal information. The image in the bottom left corner is the ground truth of sample 133 in panel d (highlighted in yellow), and notable predictions are shown for select window horizons. ( **f** ) FNO predictions based on increasing training horizons are shown for three of 77 samples of the in vitro tumor cell death data. The FNO predicts images at the 16th time point based on an input window W _i_ where 1≤i≤15. The leftmost column shows the ground truth image highlighted in yellow, and each subsequent column shows the predicted image given a window length W _i_ . Each row corresponds to a unique sample. ( **g** ) The test loss (MSE) of individual cell death images is shown as a function of training window size. Results are as variable as the other in vitro dataset. However, the spikes in test loss seem to derive from noisy backgrounds which contrast with the pure black of the ground truth. Excluding those poorly performing models, the loss curve is notably more monotonic than the cultured spheroid one. The image in the bottom left corner is the ground truth (highlighted in yellow) and notable predictions are shown for select window horizons.

## Description

Biological imaging techniques have achieved a high degree of spatial and temporal resolution, situating them as an important data source alongside ’omics data for biological discovery (Bagheri et al., 2022; Kuhn Cuellar et al., 2022). This increase in high-resolution temporal imaging introduces new opportunities and challenges for analysis to extract insights from morphological dynamics. Machine learning shows promise in going beyond feature extraction towards learning the underlying rules governing a system (Soelistyo et al., 2022; Rotem et al., 2024). A better understanding of how temporal dynamics affect the performance of machine learning models can help uncover the timing of biological processes (Bao et al., 2025; Toulany et al., 2023) and outline the performance-cost tradeoff curve for generating high temporal resolution data (Cain et al., 2024; Aceituno et al., 2025). Here, we develop a deep learning model to forecast future morphological states of three biological systems from time-series images using input sequences of varying lengths to probe how much temporal information is needed to make accurate predictions about future states. The three systems include: (i) simulated tumors generated by an agent-based model, (ii) cultured tumor spheroids, and (iii) individual tumor cell death dynamics.

Fourier neural operators (FNOs) are a unique neural network architecture developed for learning dynamics of systems governed by smooth, continuous processes, and have found success in emulating scientific partial differential equations (PDEs) (Kovachki et al., 2021). Although FNOs have shown strong performance in forecasting time-series across physical systems (Kurth et al., 2023; Li et al., 2023; Long et al., 2024), their sensitivity to temporal context, particularly with biological image data, remains an open question that impacts if and how one might characterize time-dependent biological processes. In this work, we train FNOs to predict a future state of the system (the final timepoint of the dataset), which we term the prediction horizon (P), from a preceding image sequence of variable length, W _i_ , the training horizon, where i refers to the number of time points in the training window (Fig. 1a). We hypothesized that prediction performance would increase as a function of the training window.

As expected, FNO performance increases monotonically with training window size in the *in silico* case study (Fig. 1b, c). Models trained on the simulated tumor dataset accurately predict general emergent properties, such as tumor size and location; however, more biologically relevant characteristics only appear when the model is trained with greater values of W _i_ . Specifically, for W _i_ ≤6, there is no qualitative or quantitative (measured by test loss) improvement. When W _6<i≤9_ , accurate tumor borders begin to appear. Between W _9_ and W _10_ , the test loss decreases significantly and a clear accurate shape for the tumor emerges. In cases where W _i_ >10, the predicted tumor has clear borders and the shape becomes increasingly precise with increasing W _i_ .

When the pipeline is applied to the tumor spheroid dataset, a different trend emerges. The models trained on the *in vitro* spheroids exhibit highly variant accuracy (Fig. 1d, e). Qualitatively, predictions with W _i>6_ capture finer boundary details and steadily decrease in test loss as the window size increases. However, the prediction with W _9_ deviates from this trend, exhibiting an unusually high loss. We believe this variation in performance is due to the relatively short time-scale of the image dataset and the lack of substantial morphological changes over that period. Despite variations in the loss function, a qualitative assessment of performance points to consistent improvement with increasing W _i_ , suggesting that pixel-wise loss may not be a robust metric due to non-biological variations in the background of the predicted images. Nevertheless, the FNO seems to be learning increasingly more biological features and morphological characteristics of *in vitro* tumors with increasing W _i_ .

Having assessed performance in two distinct tumor spheroid datasets, we evaluated the impact of the training window on a cancer cell death time-lapse dataset with a finer temporal resolution. This system features a distinct biological event (cell death) that is characterized by a rapid condensing of fluoresced materials. Quantitatively, the test loss (Fig. 1f, g), exhibits non-monotonicity similar to the tumor spheroid dataset. Despite fluctuations in model performance, the FNO appears to improve as the training horizon increases. The primary inconsistencies in the loss curve qualitatively align with poor prediction of the image background, rather than in the prediction of biologically relevant dynamics. Qualitatively, results indicate that prediction accuracy for biologically relevant dynamics improves with increasing W _i_ , as the FNO captures more accurate cell morphologies. This trend demonstrates that learning temporal dynamics is highly dependent on biological variance and technical noise, and that qualitative validation should be considered in concert with quantitative metrics.

In this paper, we introduce a variable training horizon pipeline using FNOs to investigate the complexity of temporal trends of several biological systems using imaging data. Our results reveal a contrast between simulated and real-world biological dynamics. The simulated tumor growth model, governed by smooth dynamics, showed a clear monotonic improvement in prediction accuracy as more temporal information was provided. However, in both the tumor spheroid and tumor cell death dynamics datasets, increasing the temporal information shows variant, non-monotonic performance landscapes. Factors that could limit the performance of data-driven models include ineffective sampling of critical transitions, biological “switches”, and timescales where dynamics can be obscured by biological stochasticity and/or technical noise. These findings align with recent work suggesting that the loss landscape for auto-regressive prediction in dynamic systems can be surprisingly uneven (Aceituno et al., 2025), which we find to be exacerbated by the nonlinear dynamics inherent in biological processes. However, models trained on denoised microscopy images obtained by image processing methods (e.g., cell segmentation) produce smoother performance landscapes despite occasional outliers. Our results highlight both a challenge and an opportunity: while data-driven methods struggle with the unpredictability of biological systems, the model failure points can be used to identify key biological transitions.

## Methods

***In silico* tumor microenvironment simulations.** Data was generated using ARCADE, an agent-based model (ABM) characterizing tumor growth in a heterogeneous and dynamic vascular microenvironment. We simulated 500 tumors under varying microenvironmental conditions over 15 days, capturing a snapshot of the *in silico* 2D-slice of tissue every 24 hours. The simulations used for this study are similar to those described in (Yu and Bagheri, 2020).

***In vitro* cancer spheroid microscopy images.** Time-lapse images of tumor spheroids monitor the effects of interface stiffness on the invasiveness of tumor development (Thi Kim Ngan Ngo, 2022). This dataset contains time-lapse microscopy of 133 tumor spheroids cultured under varying extracellular matrix stiffness and surface topographies. We trained models on snapshots of these tumors taken every 2 hours over a period of 24 hours.

***In vitro* cell death microscopy images** . A LNCaP cell line was treated with doxorubicin, a cell death–inducing compound, and time-lapse images monitored the impact of this treatment (Vicar et al., 2020). The dataset we used spans 24 hours with a frame rate 1 frame per 3 minutes, and a spatial resolution of 1.59 px/μm. We employed StarDist (Schmidt et al., 2018), a deep learning-based cell segmentation model, to identify individual cell locations. We tracked individual cell’s location across time frames through a Kalman tracker. The timing of the cell death event was determined from the fluorescence intensity over time frames where a sharp increase indicates DNA condensation during cell death. We used 1 to 15 time frames prior to the cell death event for the window horizon input and predicted the system morphology 5 frames after the cell death.

**Spatiotemporal FNO model architecture** . The FNO model was developed with a 3D architecture—2 spatial dimensions and 1 temporal dimension—to effectively capture the spatiotemporal dynamics of tumor emergence. We implemented the FNO using the neuraloperator library (Kovachki et al., 2021; Kossaifi et al., 2024), which uses the PyTorch library (Paszke et al., 2019). Hyperparameters were defined as: modes=(24, 24, 8), layers=8, width=32. Datasets were partitioned into model training (80%), validation (10%), and test (10%) sets. All images were center-cropped and down-sampled to a fixed spatial resolution of 128 × 128. Model training minimized the H1 loss using the Adam optimizer (Kingma and Ba, 2017).

**Data and code availability.** All datasets and code are open and accessible. The deep learning model source code is available on Zenodo at 10.5281/zenodo.17478675. The ARCADE ABM v2.4 source code (used to generate the synthetic tumor images) is available on Zenodo at 10.5281/zenodo.10622155. The tumor spheroid and cell death datasets were previously published (Thi Kim Ngan Ngo, 2022; Vicar et al., 2020).
